# Multi-omics analyses reveal metabolic alterations regulated by hepatitis B virus core protein in hepatocellular carcinoma cells

**DOI:** 10.1038/srep41089

**Published:** 2017-01-23

**Authors:** Qi Xie, Fengxu Fan, Wei Wei, Yang Liu, Zhongwei Xu, Linhui Zhai, Yingzi Qi, Bingyu Ye, Yao Zhang, Sumit Basu, Zhihu Zhao, Junzhu Wu, Ping Xu

**Affiliations:** 1 School of Basic Medical Sciences, Key Laboratory of Combinatorial Biosynthesis and Drug Discovery of Ministry of Education, School of Pharmaceutical Sciences, Wuhan University, Wuhan 430071, China; 2 State Key Laboratory of Proteomics, Beijing Proteome Research Centre, National Engineering Research Centre for Protein Drugs, National Centre for Protein Sciences Beijing, Institute of Radiation Medicine, Beijing 102206, China; 3 Anhui Medical University, Hefei 230032, China; 4 Departmentof Chemical & Biomolecular Engineering, National University of Singapore, 117585, Singapore; 5 Beijing Institute of Bioengineering, Beijing 100071, China; 6 Center for Pharmacometrics and Systems Pharmacology, College of Pharmacy, University of Florida, 6550 Sanger Road, Orlando, FL 32827, USA

## Abstract

Chronic hepatitis B virus (HBV) infection is partly responsible for hepatitis, fatty liver disease and hepatocellular carcinoma (HCC). HBV core protein (HBc), encoded by the HBV genome, may play a significant role in HBV life cycle. However, the function of HBc in the occurrence and development of liver disease is still unclear. To investigate the underlying mechanisms, HBc-transfected HCC cells were characterized by multi-omics analyses. Combining proteomics and metabolomics analyses, our results showed that HBc promoted the expression of metabolic enzymes and the secretion of metabolites in HCC cells. In addition, glycolysis and amino acid metabolism were significantly up-regulated by HBc. Moreover, Max-like protein X (MLX) might be recruited and enriched by HBc in the nucleus to regulate glycolysis pathways. This study provides further insights into the function of HBc in the molecular pathogenesis of HBV-induced diseases and indicates that metabolic reprogramming appears to be a hallmark of HBc transfection.

According to the estimates from the International Agency for Research on Cancer in 2008, the global burden of human cancers caused by viral infections increased to 11% of recorded cancer cases worldwide^1^. Similar to other types of oncogenic mechanisms, viral-induced oncogenesis is a multi-step process that requires the acquisition of all the cellular features responsible for the tumour phenotype, which was originally described in ‘The hallmarks of cancer’ by Weinberg in 2000 and updated in 2011^2^,^3^. Chronic hepatitis B virus (HBV) infection accounts for up to 54% of hepatocellular carcinoma (HCC) cases globally^4^. In addition, more than 240 million individuals are predicted to be chronically infected with HBV, facing a 15–40% lifetime risk of developing end-stage liver diseases (cirrhosis, liver failure and HCC), accounting for more than six million deaths per year^5^,^6^. Despite highly developed antiviral treatment options, no effective treatment is currently available for chronic hepatitis B (CHB) or the subsequent end-stage liver diseases including HCC, because the underlying mechanisms of HBV-induced HCC are still elusive.

HBV is a small enveloped DNA virus containing four overlapping reading frames: S (encoding the viral surface proteins, HBs), P (encoding the viral polymerase), X (encoding the regulatory X protein, HBx) and pre C (encoding the antigens ‘e’ and ‘c’)^7^. Reports have indicated that the association between metabolic disorders and HCC may be attributed to the effects of HBV infection and in particular the HBV-encoded proteins^8^. For instance, HBx has been shown to accelerate lipogenesis and cause hepatic steatosis through the transcriptional activation of metabolism-related genes or by mediating a series of signalling pathways^9^,^10^,^11^.

HBV core protein (HBc) is essential in the life cycle of HBV and may represent a potential therapeutic target for the treatment of HBV^12^,^13^. It has been found that the nuclear distribution of HBc is primarily associated with minor hepatitis activity, whereas the cytoplasmic distribution of HBc is related to the occurrence of chronic liver diseases^14^. Jia *et al*. reported that the expression of HBc made cells susceptible to TNF-α-induced apoptosis, indicating the pathogenic effects of HBc in liver injury during hepatitis B infection^15^. However, the involvement of HBc in the regulation of metabolism in HBV-transfected hepatocytes has not been confirmed. The identification of proteins that interact with HBc may provide insight into the function of HBc in the regulation of cell processes^16^.

Stable isotope labelling by amino acids in cell culture (SILAC), a quantitative proteomics approach to identify differentially expressed proteins^17^, has been successfully applied to investigate the regulatory network associated with specific proteins in HCC^18^,^19^,^20^. Additionally, metabolomics analysis tools such as nuclear magnetic resonance spectroscopy (NMR) are commonly utilized in the evaluation of the systematic responses to exogenous stimuli^21^,^22^. In the case of HBV infection, approaches such as metabolomics, proteomics, and molecular biology assays have been utilized to identify the central carbon metabolism, hexosamine pathway, and disruption of nucleotide synthesis in HepG2.2.15 cells^23^.

In this study, we developed a combined proteomics and metabolomics approach to identify the metabolic alterations in HepG2 HCC cells transfected with HBc. Moreover, we identified the proteins interacting with HBc by combining co-immunoprecipitation (CoIP) with mass spectrometry (MS). Among all the tested proteins, Max-like protein X (MLX) was found to be an interacting partner of HBc. Therefore, this study provides comprehensive information to enable the generation of credible hypotheses about the function of HBc in the pathogenesis of HBV-induced diseases.

## Results

### HBc overexpression in HepG2 promotes cell proliferation and migration

**Figure 1:**
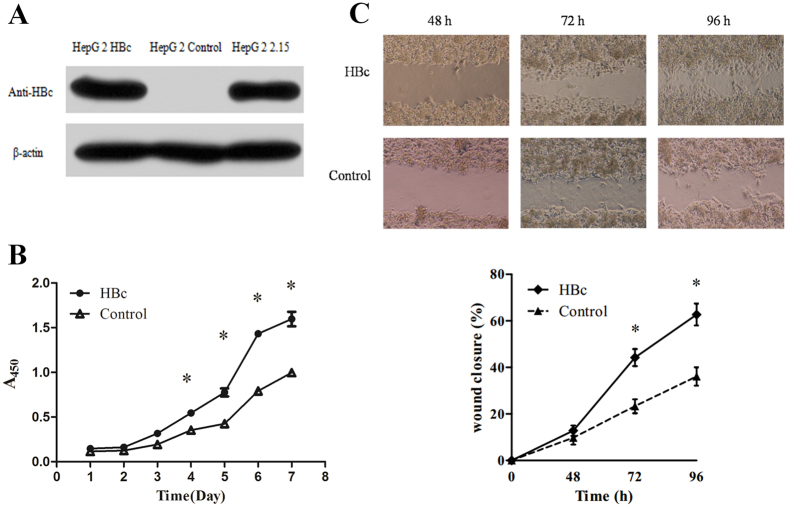
HBc promotes cell proliferation and migration. (**A**) HBc expression was detected by western blotting. (**B**) The effect of HBc on cell proliferation was measured using CCK-8 assay (*p < 0.05). (**C**) The effect of HBc on cell motility was measured using a wound-healing assay. Top: Representative photographs taken at 48 h, 72 h and 96 h post-wound (×100). Bottom: The wound closure was quantified by measuring the remaining unmigrated area using ImageJ.

The establishment of stably transfected HBc-overexpressing HepG2 cell line (HepG2-HBc) was confirmed by western blot analysis (Fig. 1A). Compared with the control cells, HBc overexpression could significantly promote cell proliferation after 4 days in culture (Fig. 1B). As shown in Fig. 1C, HBc overexpression drastically accelerated the wound-healing process after 72 h in culture, indicating that HBc might increase the migration of HepG2 cells.

### Identification of differentially expressed proteins in HBc-overexpressing cells

**Figure 2:**
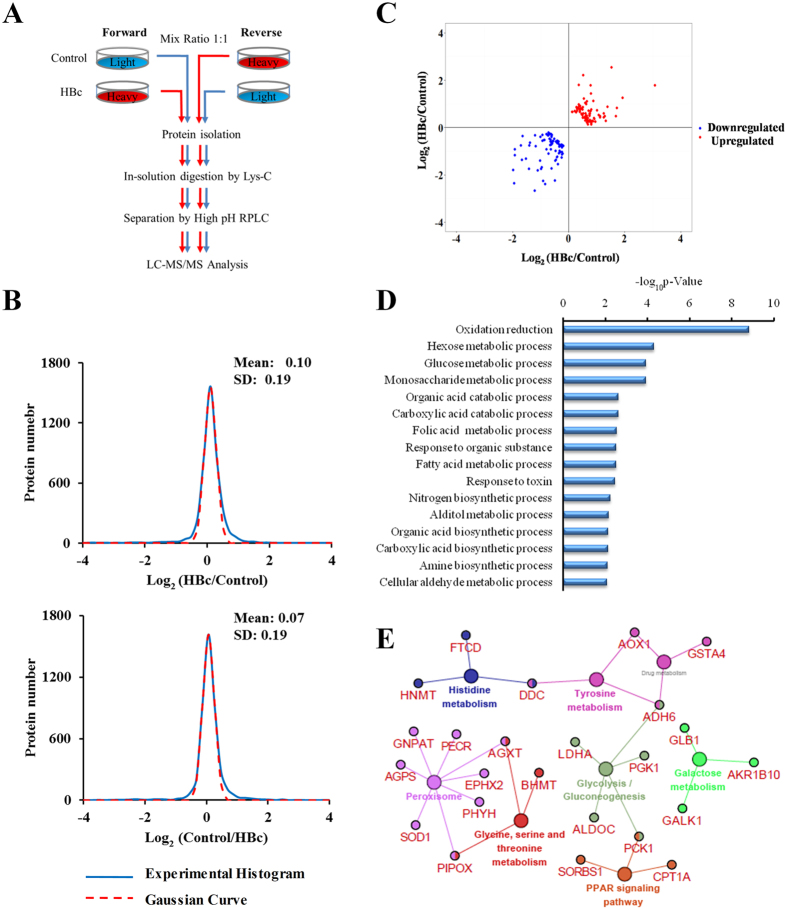
Enzymes that significantly change between HBc and control cells. (**A**) Workflow of the SILAC-based proteomic identification process with forward and reverse labelling experiments. (**B**) The quality of SILAC labelling was determined by observing the normalized log2 ratio distribution of the differentially expressed proteins from the forward and reverse datasets. (**C**) The scatter plot displays the regulated proteins based on the protein expression ratios of HBc and control cells. (**D**) Functional enrichment analysis of differentially expressed proteins. The y-axis presents the functional categories identified in the GO analysis in terms of biological processes. The x-axis demonstrates the significance (*p* < 0.05). (**E**) KEGG annotation revealed the networks up-regulated by HBc. Large nodes represent metabolites within core regulatory networks. Enzymes are represented by small nodes. The results only showed pathways with *p* < 0.05 and cluster protein number ≥ 3.

To investigate the biological function of HBc, we screened the differentially expressed proteins induced by HBc overexpression using a SILAC-based quantitative proteomic strategy. A schematic diagram illustrating the experimental design is presented in Fig. 2A. A total of 5016 and 4998 proteins were identified in the forward and reverse labelling experiments, respectively. Among those proteins, a total of 4264 and 4257 proteins were quantified in the forward and reverse labelling experiments, respectively, of which 3734 proteins were overlapped. Histogram plots of the log2 ratio distributions of the quantified proteins in each labelling experiment were fit to Gaussian curves with the same standard deviation (SD) of 0.19 (Fig. 2B). By setting a 3-fold SD value as the threshold, a total of 165 differentially expressed proteins were detected, of which 84 proteins were up-regulated and 81 were down-regulated in HBc-overexpressing cells (Fig. 2C and Table S1).

According to the Gene Ontology (GO) analysis, the up-regulated proteins were predominantly related to the metabolism of carbohydrates (hexose, glucose and monosaccharide metabolic processes), organic acid catabolic processes, and fatty acid metabolic processes (Fig. 2D). The ClueGO plug-in Cytoscape (v 3.3.0) was used to further perform a KEGG pathway analysis, which enables the comparison of clusters/groups to highlight their functional specificity. These analyses indicated that the HBc up-regulated proteins were mainly associated with the alteration of metabolic pathways, including glycolysis and glycine and tyrosine metabolism (Fig. 2E). However, the HBc down-regulated proteins did not show such a clear trend of metabolic clustering (Fig. S1). Together, these results indicate that HBc may accelerate the processes of cell metabolism.

### Metabolic characterization of HBc-overexpressing cells

**Figure 3:**
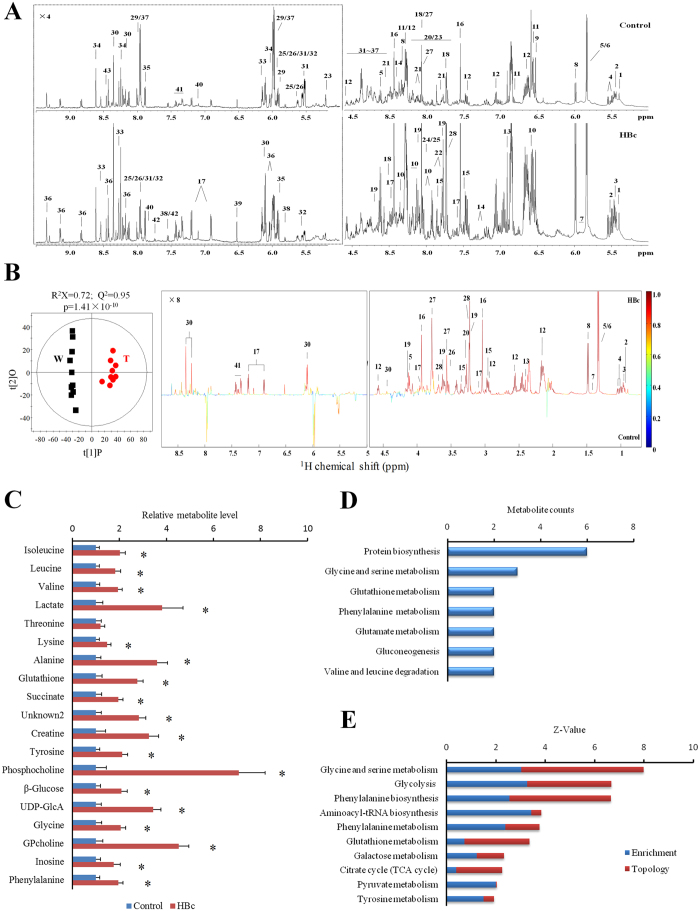
Metabolic characterization of HBc cells. (**A**) Average 600 MHz ^1^H HRMAS NMR spectra of HBc and control cells. The region of δ5.0–9.5 was vertically expanded 4 times compared with δ0.5–4.5. The metabolite list is shown in Table S2. (**B**) Validated OPLS-DA scores and coefficient plots show the discriminations of the metabolic profiles of extracts of HBc cells (T) from control cells (W). The resonance peaks pointing upward indicate an increase of metabolites in HBc cells and vice versa. The colour of the peaks represents the correlation coefficient of a metabolite where the cut-off value of |*r*| was 0.847 (n = 10, *p* < 0.001). The detailed data are shown in Table S2. (**C**) Metabolites consumed by HBc and control cells. The data are shown as the mean ± standard deviation (SD), n = 1 0, *t* test, *p < 0.001. Notes: UDP-GlcA, UDP-glucose A; GPcholine, glycerophosphocholine. (**D**) The enrichment analysis displays the metabolic pathways regulated by HBc. (**E**) The integrated metabolic pathways were obtained from metabolomics and proteomics analyses.

A metabolomics study was conducted to verify the potential metabolic changes indicated by the quantitative proteomics data from HBc-overexpressing cells. The average ^1^H NMR spectra of both HBc-overexpressing and control cells (Fig. 3A) revealed a number of metabolites that were unambiguously assigned based on the literature data, which were further confirmed by a series of 2D NMR spectral data (Table S2). There were significant differences in the metabolite changes between the HBc-overexpressing and control cells based on the OPLS-DA model. A further CV-ANOVA analysis confirmed the statistical significance of these data (*p* < 0.05). HBc-overexpressing cells contained relatively higher levels of lactate, glutathione (GSH), phosphocholine (PC), glycerophosphocholine (GPC) and a range of amino acids such as glycine, tyrosine and phenylalanine (Fig. 3B,C), compared with control cells.

According to a metabolite set enrichment analysis by MetaboAnalyst 3.0, these HBc up-regulated metabolites were mainly related to protein biosynthesis, gluconeogenesis, and glycine and phenylalanine metabolism (Fig. 3D). Additionally, we systematically calibrated the proteomics and metabolomics data using the same software. The highlighted representation pathways were glycine metabolism, glycolysis, and phenylalanine and tyrosine biosynthesis (Fig. 3E), further confirming that HBc has the ability to promote cellular metabolic processes.

**Figure 4:**
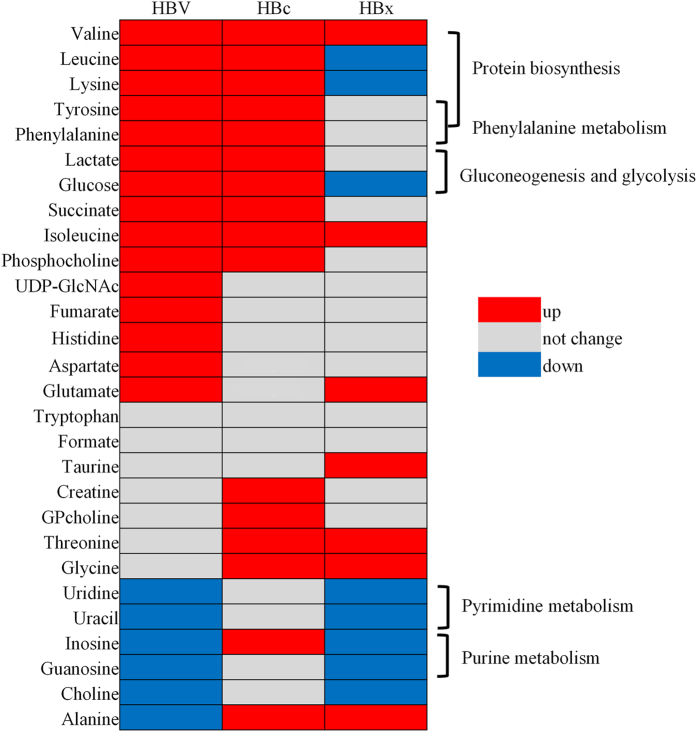
Comparison of metabolic patterns between HBV and its encoded genes. The metabolomics datasets were collected from our study and published papers^23^,^24^. The metabolites jointly identified by these studies were selected and analysed here. The significantly up- or down-regulated metabolites (*p < 0.001) are indicated by red or blue, respectively.

To validate that HBc may contribute to HBV-related metabolism, we reanalysed and compared recently published metabolomics researches involving HBV^23^ and HBx^24^ transfection models. Those studies mentioned above used the same control HepG2 cells as this study and compared them with either HBV-infected HepG2 cells or HepG2 cells transfected with the full-length HBx gene, respectively. More importantly, all three metabolomics datasets were collected on a Bruker AV III 600 MHz spectrometer, confirming a similar background for these data. These features facilitated the subsequent analysis and comparison. A systematic analysis showed that 28 metabolites were overlapped (Fig. 4), 13 of which exhibited the same trend between HBV-infected and HBc-transfected cells (10 co-up-regulated and 3 did not change), whereas between HBV-infected and HBx-transfected cells, 12 metabolites presented the same trend (3 co-up-regulated, 4 did not change and 5 co-down-regulated). A further clustering analysis found that the 10 co-up-regulated metabolites induced by either HBc transfection or HBV infection were mainly related to protein synthesis, phenylalanine metabolism, and the gluconeogenesis pathway (*p* < 0.05), which is consistent with our previous result (Fig. 3D). By contrast, the HBx and HBV co-down-regulated metabolites were related to purine and pyrimidine metabolism, which is also consistent with the documented HBx function^24^.

The inconsistency of metabolomics profiles may be attributed to the different expression of the enzymes in metabolic pathways. Fumarate was up-regulated in HBV transfected cells, but not changed in HBc or HBx transfected cells. Correspondingly, the expression of succinate dehydrogenase (SDHB), which is responsible for fumarate synthesis from succinate, was up-regulated in HBV transfected cells (Table S4B in ref. 23), but not changed in HBc or HBx transfected cells. Similarly, histidine was up-regulated in HBV transfected cells, but not changed in HBc overexpression cells due to the enzyme–Formimidoyltransferase-cyclodeaminase (FTCD), as a part of the pathway of L-histidine degradation into L-glutamate, was up-regulated in HBc transfected cells (Fig. 2E).

These data suggested that HBc contributes to HBV-related metabolism dysregulation through the modulation of glycolysis and amino acid metabolism, which is apparently differentiable from the HBx-induced altered nucleic acid metabolic pathway.

### HBc mediates glycine and phenylalanine pathway activation by directly binding with enzymes of the pathway

**Figure 5:**
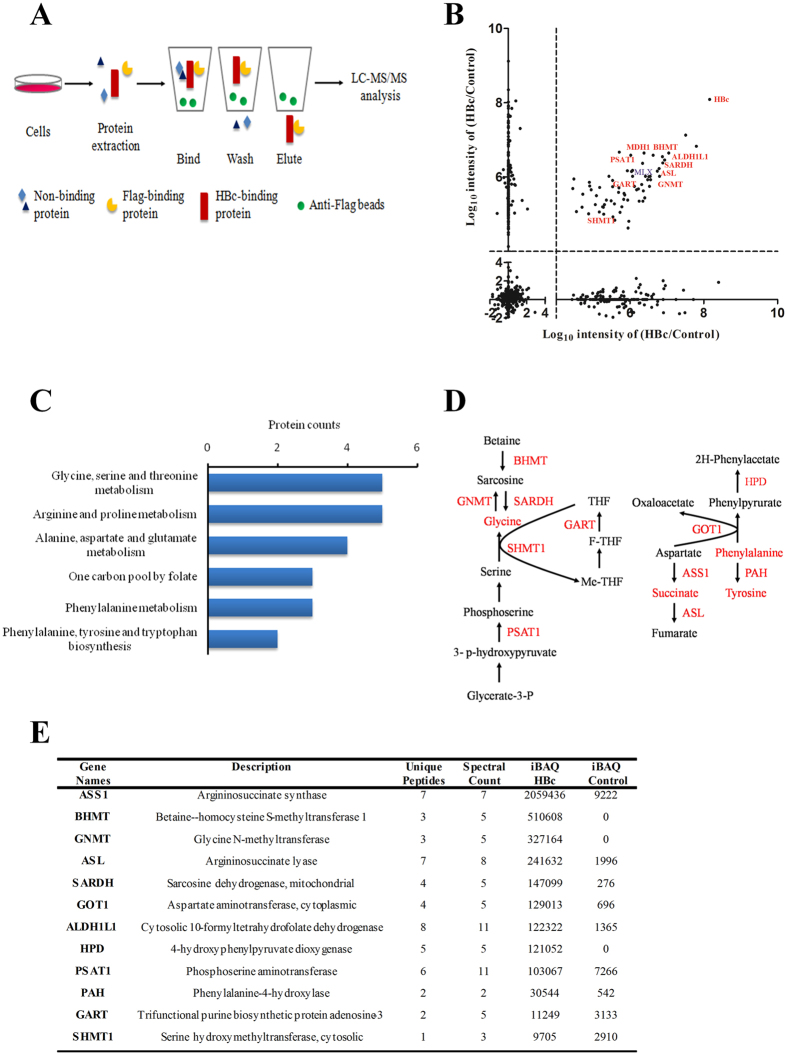
Identification of the interacting proteins of HBc. (**A**) A schematic diagram of the CoIP experiment for enriching the HBc interacting proteins. (**B**) The distribution of potential HBc interacting proteins is shown in the scatter plot with a cut-off transformed log10 intensity > 4. The highlighted dots represent some potential candidates in this study. (**C**) The KEGG annotation revealed the pathways regulated by HBc. (**D**) Graphical demonstration of the predominant enzymes that bind HBc and their involvement in the metabolic pathways. (**E**) MS information of the HBc interacting proteins.

Co-immunoprecipitation coupled with LC-MS/MS was employed to identify putative interacting proteins of HBc. A schematic diagram of the experiment is shown in Fig. 5A. To enhance the accuracy and reliability of the experiment, we analysed two independent biological duplicates. A higher intensity ratio compared with the control (Fig. 5B and Table S3) represented the potential interacting proteins of HBc with high confidence (log10 intensity of HBc/control > 4). To highlight these data, some key proteins have been marked in red.

Interestingly, the KEGG pathway analysis indicated that these potential HBc binding proteins were predominantly related to glycine metabolism and phenylalanine and tyrosine biosynthesis (Fig. 5C), which was consistent with the proteomic and metabolomic findings above (Fig. 3E). To further verify that HBc directly binds with enzymes in the metabolic pathways, we manually checked the MS data of the proteins listed in the KEGG pathway (Fig. 5D,E). These results suggested that HBc could directly bind with the enzymes in glycine metabolic pathways (Fig. 5D, left), such as glycine N-methyltransferase (GNMT) and sarcosine dehydrogenase (SARDH). These enzymes regulate the conversion process between glycine and sarcosine, which contributes to the activation of glycine metabolic pathways. Similarly, HBc could also directly interact with the enzymes involved in phenylalanine degradation (Fig. 5D, right), such as phenylalanine hydroxylase (PAH) and 4-hydroxyphenylpyruvate dioxygenase (HPD), which are involved in different steps of the sub-pathway that synthesizes acetoacetate and fumarate from phenylalanine. The MS information of all the enzymes mentioned above are summarized in Fig. 5E. These results led to the conclusion that HBc could interfere with glycine and phenylalanine metabolism by directly binding to enzymes in the responsible pathways.

### HBc facilitates the activation of the glycolytic pathway via MLX

**Figure 6:**
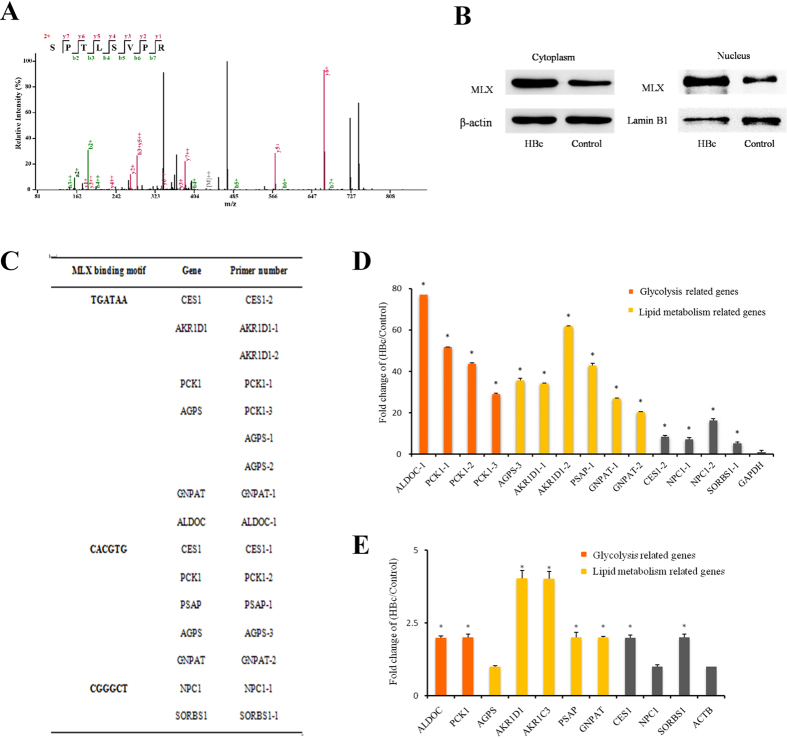
Identification of the transcriptional regulation effect of MLX. (**A**) The MLX relative abundance was represented by the monoisotopic m/z values of the detected peptide of MLX. (**B**) MLX expression was detected by western blotting. (**C**) Primers were designed according to MLX binding motifs. (**D**) ChIP-qPCR results showed that MLX regulated the expression of differentially expressed proteins (*p < 0.001). (**E**) The expression of MLX-regulated genes was detected by real-time PCR (*p < 0.05).

The above results demonstrate that HBc directly regulates the metabolism of amino acids. However, the mechanism by which HBc regulates glucose and lipid metabolism still requires clarification. Further analysis of the interaction of proteins with HBc indicated that MLX, as a transcription factor, might regulate carbohydrate and lipid metabolism. The spectra quality of MLX was manually checked, and the specific peptide was found with high base peak intensity and continuous b/y ion matches (Fig. 6A), demonstrating the confidence of the MLX identification. Subsequently, immunoblot analysis was used to compare the expression levels of MLX in the cytoplasm and nucleus between HBc-overexpressing cells and control cells. The results showed that HBc could increase the expression of MLX, especially in the nucleus (Fig. 6B), which thus indicated that MLX might be recruited and enriched by HBc in the nucleus to regulate the relevant signalling pathways.

To identify the specific substrates regulated by MLX in the HBc-overexpressing cells, ChIP assays and qPCR were performed. In HBc-overexpressing cells, the MLX protein bound to an upstream regulatory sequence in the aldolase C (ALDOC) gene via the TGATAA motif (Fig. 6C,D) and promoted its expression (Fig. 6E). Additionally, enhanced binding was observed between MLX and the upstream regulatory sequence of phosphoenolpyruvate carboxykinase (PCK1), which contains 3 motifs (2 TGATAA motifs and 1 CACGTG motif) (Fig. 6C,D). These results indicated that the key enzymes ALDOC and PCK1 in the glycolytic pathway were both up-regulated by MLX following HBc transfection (Fig. 6E). Similarly, our ChIP-qPCR results showed that HBc overexpression enhanced the effect of MLX on the transcription regulation of aldo-keto reductase family 1 member D1 (AKR1D1), dihydroxyacetone phosphate acyltransferase (GNPAT) and prosaposin (PSAP) (Fig. 6D,E), which indicates the possible involvement of MLX in cellular lipid metabolism.

### Major metabolic modules are rewired by HBc

**Figure 7:**
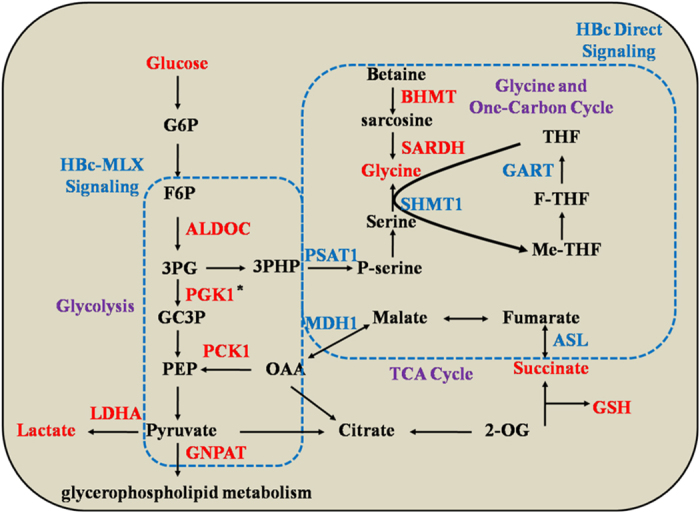
Summary of the variation of metabolic pathways induced by HBc. Metabolites and enzymes shown in red indicate higher expression levels and direct interaction with HBc or MLX. Enzymes without significant changes are marked in blue. *PGK1 expression was significantly increased in the proteomics data, but the interaction between PGK1 and MLX is still unclear. Abbreviations: G6P, glucose-6-phosphate; F6P, fructose-6-phosphate; 3 PG, glyceraldehyde-3-phosphate; GC3P, 3-phosphoglyceric acid; PEP, phosphoenolpyruvate; OAA, oxaloacetate; 3PHP, 3-phosphohydroxypyruvate; P-serine, phosphatidylserine; 2-OG, 2-oxoglutarate; THF, tetrahydrofolate; me-THF; 5,10-methylene-THF; F-THF, 10-formyltetrahydrofolate; GSH, glutathione.

The final list of predicted enzymes associated with HBc transfection-induced metabolic pathways dysregulation spanned glycolysis, glycine metabolism and the tricarboxylic acid cycle (TCA) (Fig. 7). The expression of predicted targets were significantly promoted in HBc-overexpressing cells. On top of that, the changes in the metabolic pathways are induced by two primary mechanisms. On one hand, HBc could directly bind to the enzymes of the glycine metabolic pathway, including betainehomocysteine S-methyltransferase (BHMT) and sarcosine dehydrogenase (SARDH). On the other hand, it could regulate the glycolysis pathway by increasing the expression of MLX, followed by the up-regulation of the key enzymes ALDOC and PCK1.

## Discussion

In this study, the metabolic features associated with HBc overexpression were identified through the integration of proteomics, metabolomics, CoIP-MS, and ChIP-qPCR assays. The results demonstrated that HBc is able to modify the metabolic characteristics of HCC cells. In future studies, we are going to explore the role of HBc in the metabolic pathways of amino acid metabolism and glycolysis to decipher the molecular pathological mechanisms of HBV infection.

Over the past several years, the idea has emerged that the progression of cancer involves major alterations in cell metabolism. An increased rate of glycolysis and synthesis of fatty acids are salient features of different types of cancers. Cell proliferation and metabolism are tightly coupled with the cellular processes^25^,^26^. As intracellular parasites, viruses depend on the metabolism of infected cells to proliferate. To avoid metabolic exhaustion, viruses manipulate metabolic pathways and associated signalling cascades to provide sufficient resources to support the optimal production of virions, which indicates that metabolic reprogramming is a hallmark of viral oncogenesis^27^. Copeland *et al*. demonstrated that a large proportion of genes that drove tumourigenesis in a mouse model of HBV-induced HCC were associated with aerobic glycolysis and anabolic metabolic processes^28^. In this study, we found that HBc promoted the expression of metabolic enzymes and the secretion of metabolites in HCC cells. Surprisingly, our results argue that HBc contributes to HBV-related metabolic dysregulation through the modulation of glycolysis and amino acid metabolism, which is significantly different from the metabolic changes in nucleic acid metabolism induced by HBx^23^,^24^. Meanwhile, a recent study showed that the majority of amino acids (glycine, phenylalanine, glutamic acid, asparagine and tyrosine) were considerably up-regulated in HBV patients, compared with healthy subjects^29^. This study concluded that 9 pathways were considered closely related to the development of HCC, including aminoacyl-tRNA biosynthesis and phenylalanine and glycine metabolism. The metabolic characteristics of HBV-related HCC patients would strengthen our understanding of the role of HBc in the proliferation and migration of tumour cells.

A large-scale study quantified the consumption and release profiles of 219 metabolites from media across the NCI-60 cancer cell lines. The results indicated that the consumption of glycine and the expression of the mitochondrial glycine biosynthetic pathway were positively correlated with rates of proliferation across cancer cell lines^30^. In our study, HBc not only regulated glycine metabolites expression but also directly affected the activity by binding with enzymes in the glycine metabolic pathway. Previous studies have found that these enzymes might be promising biomarker candidates for HCC^31^ and non-alcoholic fatty liver disease^32^. However, whether these enzymes play important roles in HBV-related HCC requires further study.

Moreover, MAX- and MLX-centred transcription networks are vital in metabolic homeostasis in normal and neoplastic cells^33^. Knockdown of MLX to blocks the Myc-induced reprogramming process of metabolic pathways and apoptosis, suggesting the significance of MLX in glycolysis and lipid biosynthesis in tumourigenesis^34^. Our results showed that HBc could increase the expression of MLX. Additionally, ALDOC and PCK1, the key enzymes in the glycolytic pathway, were up-regulated by MLX, which may be a potential pathway regulated by HBc. Both the transcription and translation of PCK1 have been reported to be up-regulated by hepatitis C virus infection, which clearly indicates its impact on hepatic glycolysis^35^. As expected, PGK1 was up-regulated by HBc in our study. A recent study highlighted that PGK1 acted as a protein kinase in coordinating glycolysis and the TCA, which is instrumental in cancer metabolism and tumourigenesis^36^. These results are consistent with a previous study indicating that PGK1 may be a promising biomarker for the progression of HCC^37^.

However, there are still several limitations in this study. Proteomics and metabolomics still cannot completely quantify and identify all the proteins and metabolites *in vivo*. Therefore, more advanced and sensitive high-throughput omics technologies are required to describe the entire regulatory network. For example, the genomics study might provide information regarding the basis accounts for the alterations in metabolomics and proteomics. In a previous study, whole-genome chromatin immunoprecipitation microarray (ChIP-on-chip) analysis was used to identify HBc-bound human gene promoters. The results showed that HBc bound to a large number of human gene promoters to mediate primary metabolic processes^38^. Furthermore, Copy-number variations (CNVs) were significantly increased at HBV breakpoint locations where chromosomal instability was likely induced. HBx, HBs and HBc could integrate with human genes to influence patient survival^39^,^40^. In addition, hot spot mutations in HBc (L60V, S87G and I97L) can affect HBV replication, viral persistence, and immunopathogenesis during chronic viral infection^41^,^42^. Thus, we are preparing to integrate the genomics data and transcriptomics data to study the role of HBc in the HBV-HCC.

In conclusion, this study demonstrates that HBc promotes the expression of metabolic enzymes and the secretion of metabolites in HCC cells, particularly activating the glycolysis and amino acid metabolism pathways. Clinical evidence has linked cell metabolism with cancer prognosis, which has raised interest in targeting metabolic enzymes for cancer therapy^43^. Therefore, our study provides new insights into the molecular pathogenesis associated with chronic HBV infection as well as the exploration of potential new therapeutic targets in HBV-related HCC.

## Materials and Methods

### Plasmid vectors, cell lines and transfection

HBc coding sequence was cloned into the plasmid vector p3 × Flag-CMV-14 (Sigma, St. Louis, MO, USA) to obtain the HBc-expressing plasmid p3 × Flag-CMV-14/HBc. The HCC cell line HepG2 was received from Professor Shan Cen at the Institute of Medicinal Biotechnology, Chinese Academy of Medical Sciences. The cells were maintained in DMEM (HyClone, Logan, UT, USA) supplemented with 10% (V/V) FBS (Gibco, Carlsbad, CA, USA) at 37 °C in an atmosphere of 5% CO_2_ with saturated humidity. The HepG2 cells were transfected with either the HBc-expressing plasmid p3 × Flag-CMV-14/HBc or the control vector p3 × Flag-CMV-14 using the FuGene HD transfection reagent (Roche, Basel, Switzerland). The stable HBc-overexpressing cells (HepG2-HBc) and control cells (HepG2-3 × Flag) were established by culturing the cells in the presence of G418 selection pressure. The transfection efficiency was determined by western blotting.

### Cell proliferation and wound-healing assay

Cell proliferation rates were examined by the CCK8 colorimetric assay according to the manufacturer’s instructions (Dojindo Laboratories, Tokyo, Japan). A wound-healing assay was performed to evaluate the cell motility. Briefly, HepG2-HBc and HepG2-3 × Flag cells were seeded in a 6-well plate. When the cells reached approximately 80–90% confluency, a pipette tip was used to make a straight scratch to simulate a wound. The remaining cells were cultured routinely, and the wound closure was monitored and photographed using an inverted microscope equipped with a camera at regular time intervals (Olympus, Tokyo, Japan).

### Sample preparation and MS Analysis

HepG2-HBc and HepG2-3 × Flag cells were maintained in SILAC DMEM (HyClone, Logan, UT, USA) supplemented with 10% dialysed FBS and either light [^12^C_6_]-L-lysine or heavy[^13^C_6_]-L-lysine (Cambridge Isotope Laboratories, Tewksbury, MA, USA) for stable isotope labelling. Equal amounts of protein from HepG2-HBc/HepG2-3 × Flagcells ([^12^C_6_]-L-lysine) and HepG2-3 × Flag/HepG2-HBc cells ([^13^C_6_]-L-lysine) were mixed and resolved by 10% SDS-PAGE. Each lane was cut into approximately 1 mm^3^ and digested with trypsin. The digested peptides were identified using an LTQ-OrbitrapVelos instrument (Thermo Fisher Scientific, San Jose, CA, USA). All MS and MS/MS raw data were analysed and processed with MaxQuant (version1.4.7) for peptide identification and quantification^44^. The Andromeda search engine was run against the NCBI database (Refseq20121107)^45^. The maximal mass tolerance in MS mode was set to 20 ppm for the first search and 6 ppm for the main search, and that in MS/MS mode was set to 0.7 Da. The maximum false discovery rates (FDR) for peptide and protein identifications were specified as 0.01.

### Extraction of the intracellular metabolites and NMR spectral data analysis

The cells were harvested by trypsinization, washed with cold PBS 3 times, and then resuspended in 1 mL of water/methanol solution (1:2, v/v), followed by three freeze-thaw cycles. The supernatants were collected after sonication and centrifugation. The remaining pellets were further extracted twice using the same procedure. The supernatants collected were pooled together and centrifuged at 12000× g at 4 °C for 10 min. The supernatant was lyophilized after the removal of methanol by SpeedVac and stored at −80 °C until NMR analysis. All 1D ^1^H NMR spectra of the cell extracts and media were acquired by a Bruker AVIII 600 MHz NMR spectrometer equipped with a cryogenic probe (Bruker Biospin, Bremen, Germany). Each spectrum was corrected for phase and baseline deformation manually using Topspin 3.0 (Bruker Biospin), and the chemical shift of TSP was calibrated at δ0.00. The normalized data were used for multivariate analysis, and the model was constructed using the orthogonal projection to latent structure-discriminant analysis (OPLS-DA) with Pareto variance (Par) scaling and validated with a 7-fold cross-validation method using SIMCA-P+ (v12.0,Umetrics, Sweden)^46^. The significance of the model was also validated by a CV-ANOVA method (*p* < 0.05)^47^. To facilitate the biological interpretation of the loadings generated from the model, the loadings were first back-transformed and then plotted with colour-coded OPLS-DA coefficients in MATLAB 7.1. The colour code corresponds to the absolute value of the OPLS-DA correlation coefficients (|*r*|), which indicated the contribution of the corresponding variable to the group separation. A value of |*r*| greater than 0.847 was considered to be significant (n = 10, *p* < 0.001).

### Bioinformatics and statistical analyses

The quantitative information of differentially expressed proteins derived from SILAC proteomics was used to perform functional classification by GO analysis in terms of biological processes^48^. Further GO enrichment analysis was processed by the ClueGO plug-in of Cytoscape 3.3.0, which integrates GO terms as well as KEGG pathways and creates a functionally organized GO/pathway term network^49^,^50^. MetaboAnalyst3.0 51 was used for the metabolite set enrichment and metabolic pathway analysis. In addition, we used this software to conduct a full analysis of the data from the proteomics and metabolomics experiments. The main function of this module is to pinpoint the pathways involved in the underlying biological processes by combining the evidence based on alterations in both protein expression and metabolite concentrations. Moreover, a topology analysis was used to evaluate the relative importance of the proteins/compounds based on their relative locations. All the results are shown as the means ± SDs. The significance of the difference in means was determined by a *t* test.

### Co-immunoprecipitation and mass spectrometry analysis (CoIP-MS)

Cells were washed 3 times with cold PBS and disrupted in lysis buffer for 2 h. The cell lysates were centrifuged at 3500 × g for 10 min, and the supernatants were further centrifuged at 17000× g for 20 min. Then, the supernatants were incubated with an anti-Flag antibody (Sigma, St. Louis, MO, USA) for 2 h. Next, 30 μL of a protein A/G agarose bead suspension (Thermo Fisher Scientific, Rockford, IL, USA) was added to the mixture and then rotated for 2 h at 4 °C. The agarose beads were collected after centrifugation and incubated with 100 μL of 1 × SDS loading buffer and 10 mM DTT for 5 min at 95 °C, followed by a quick chill on ice. The products were incubated in the dark with 50 mM IAA for 30 min at 4 °C, and the co-immunoprecipitates were collected by centrifuging at 12000× g for 5 min at 4 °C. The precipitates from both HBc cells and control cells were subjected to SDS-PAGE and in-gel digestion, followed by LC-MS/MS analysis as described above.

### Western blot analysis

Protein was extracted from cultured cells using RIPA lysis buffer (Sigma, St. Louis, MO, USA). In total, 30–50 μg of protein was resolved by 10% SDS-PAGE and transferred to nitrocellulose membranes. The blots were probed with 1:1000 diluted primary antibodies specific for HBc (Abcam, Cambridge, UK), β-actin (Abcam, Cambridge, UK), MLX (Santa-Cruz Biotechnology, Santa Cruz, CA, USA) or lamin B1 (Proteintech, Chicago, IL, USA) overnight at 4 °C, followed by anti-mouse secondary antibodies. The protein bands were then visualized using an enhanced chemiluminescence image analyser (Tanon, Shanghai, China).

### ChIP-qPCR assays

The cells were treated with 1% formaldehyde for 15 min at room temperature, harvested in lysis buffer and sheared to produce DNA fragments of ~500 bps. The samples were immunoprecipitated with beads and subsequently washed and eluted by a chromatin immunoprecipitation kit following the manufacturer’s instructions (Sigma, St. Louis, MO, USA). The cross-linked products were removed at 65 °C for 4 h, and the immunoprecipitated DNA was purified (Qiagen, Düsseldorf, Germany) and amplified by polymerase chainreaction. By searching the Transcriptional Regulatory Element Database^52^, we found that MLX bound to upstream regulatory sequence motifs containing 5′-CACGTG-3′,5′-TGATAA-3′ and 5′-CCGGAAGT-3′. Accordingly, we designed primers against motifs 0-5000 bps before the initiation regions of target genes. The sequences of these primers are presented in Fig. 6C and Table S4. The primers used for qPCR were designed using the Primer 6.0 software (Table S5).

## Additional Information

**How to cite this article**: Xie, Q. *et al*. Multi-omics analyses reveal metabolic alterations regulated by hepatitis B virus core protein in hepatocellular carcinoma cells. *Sci. Rep.* **7**, 41089; doi: 10.1038/srep41089 (2017).

**Publisher's note:** Springer Nature remains neutral with regard to jurisdictional claims in published maps and institutional affiliations.

## Supplementary Material

Supplemental Figures and Tables
