# Supplementary material for: Multi-omics analyses reveal metabolic alterations regulated by hepatitis B virus core protein in hepatocellular carcinoma cells
Source: Sci Rep. 2017 Jan 23;7:41089. doi: 10.1038/srep41089 (PMC5253728; doi:10.1038/srep41089)
Supplement: Supplemental Figures and Tables [file srep41089-s1.pdf]

Multi-omics analyses reveal metabolic alterations regulated by hepatitis B virus core protein in hepatocellular carcinoma cells

Qi Xie[1,2ǂ], Fengxu Fan[2,3ǂ], Wei Wei[2ǂ], Yang Liu[4ǂ], Zhongwei Xu[2], Linhui Zhai[2], Yingzi Qi[2], Bingyu Ye[5], Yao Zhang[2],Sumit Basu[6],Zhihu Zhao[5], Junzhu Wu[1 *], Ping Xu[1,2,3 *]

[1]School of Basic Medical Sciences, Key Laboratory of Combinatorial Biosynthesis and Drug Discovery of Ministry of Education, School of Pharmaceutical Sciences, Wuhan University, Wuhan 430071, China;

[2]State Key Laboratory of Proteomics, Beijing Proteome Research Centre, National Engineering Research Centre for Protein Drugs, National Centre for Protein Sciences Beijing, Institute of Radiation Medicine, Beijing 102206, China;

[3]Anhui Medical University, Hefei 230032, China;

[4]Departmentof Chemical & Biomolecular Engineering, National University of Singapore, Singapore 117585;

[5]Beijing Institute of Bioengineering, Beijing 100071, China;

[6]Center for Pharmacometrics and Systems Pharmacology, College of Pharmacy, University of Florida, 6550 Sanger Road, Orlando, FL 32827, USA.

ǂ These authors contributed equally to this work.

*Corresponding authors,

Ping Xu, Beijing Proteome Research Center, 38 Science Park Road, Changping District, Beijing 102206, China

Tel: +8610-61777113, Fax: +8610-61777050, Email: xuping@mail.ncpsb.org

Junzhu Wu,185 Donghu Road, Wuchang District, Wuhan 430071, China

Tel: +8627-68759311, Fax: +8627-68759222, Email: wujunzhu@whu.edu.cn

Supplementary Figure Legends

Figure S1. Molecular Function enrichment revealed the network down-regulated by HBc. The results only showed pathways with $P<0.05$ and cluster protein number $\geqslant 3$.

Figure S2. Sequence alignment results of the HBc recombinant plasmid.

Figure S1

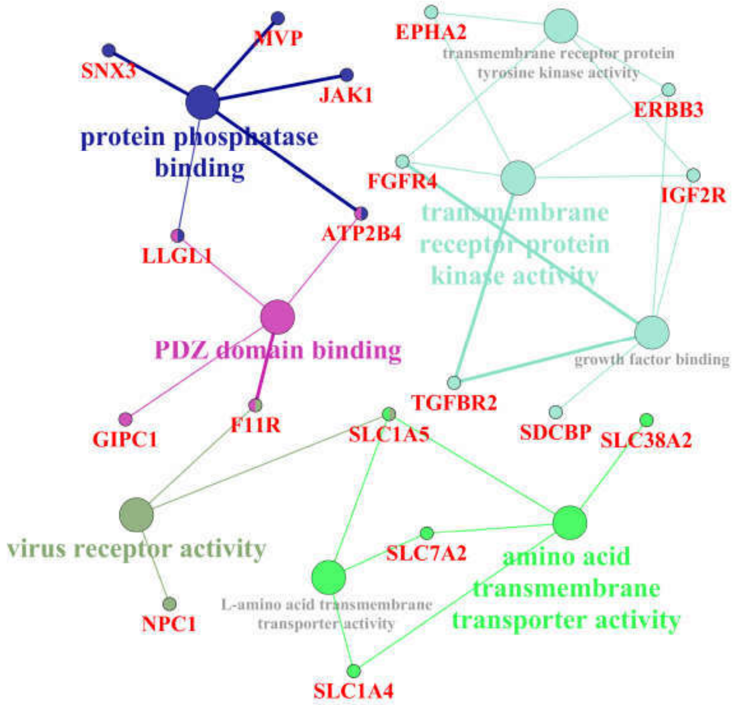

MVP
SNX3
JAK1
protein phosphatase binding
EPHA2
transmembrane receptor protein tyrosine kinase activity
ERBB3
FGFR4
IGF2R
transmembrane receptor protein kinase activity
ATP2B4
LLGL1
PDZ domain binding
growth factor binding
TGFBR2
GIPC1
F11R
SLC1A5
SDCBP
SLC38A2
virus receptor activity
SLC7A2
amino acid transmembrane transporter activity
L-amino acid transmembrane transporter activity
NPC1
SLC1A4

Figure S2

HBC-3FLAG.CMV-F(H03)	ATGCAACTTTTTCACCTCTGCCTAATCATCTCTTGTTCAT	79
HBC_ORF	ATGCAACTTTTTCACCTCTGCCTAATCATCTCTTGTTCAT	40
Consensus	atgcaactttttcacctctgcctaatcatctcttgttcat	
HBC-3FLAG.CMV-F(H03)	GTCCTACTGTTCAAGCCTCCAAGCTGTGCCTTGGGTGGCT	119
HBC_ORF	GTCCTACTGTTCAAGCCTCCAAGCTGTGCCTTGGGTGGCT	80
Consensus	gtcctactgttcaagcctccaagctgtgccttgggtggct	
HBC-3FLAG.CMV-F(H03)	TTGGGGCATGGACATCGACCCTTATAAAGAATTTGGAGCT	159
HBC_ORF	TTGGGGCATGGACATCGACCCTTATAAAGAATTTGGAGCT	120
Consensus	ttggggcatggacatcgacccttataaagaatttggagct	
HBC-3FLAG.CMV-F(H03)	ACTGTGGAGTTACTCTCGTTTTTGCCTTCTGACTTCTTTC	199
HBC_ORF	ACTGTGGAGTTACTCTCGTTTTTGCCTTCTGACTTCTTTC	160
Consensus	actgtggagttactctcgtttttgccttctgacttctttc	
HBC-3FLAG.CMV-F(H03)	CTTCAGTACGAGATCTTCTAGATACCGCCTCAGCTCTGTA	239
HBC_ORF	CTTCAGTACGAGATCTTCTAGATACCGCCTCAGCTCTGTA	200
Consensus	cttcagtacgagatcttctagataccgcctcagctctgta	
HBC-3FLAG.CMV-F(H03)	TCGGGAAGCCTTAGAGTCTCCTGAGCATTGTTCACCTCAC	279
HBC_ORF	TCGGGAAGCCTTAGAGTCTCCTGAGCATTGTTCACCTCAC	240
Consensus	tcgggaagccttagagtctcctgagcattgttcacctcac	
HBC-3FLAG.CMV-F(H03)	CATACTGCACTCAGGCAAGCAATTCTTTGCTGGGGGGAAC	319
HBC_ORF	CATACTGCACTCAGGCAAGCAATTCTTTGCTGGGGGGAAC	280
Consensus	catactgcactcaggcaagcaattctttgctggggggaac	
HBC-3FLAG.CMV-F(H03)	TAATGACTCTAGCTACCTGGGTGGGTGTTAATTTGGAAGA	359
HBC_ORF	TAATGACTCTAGCTACCTGGGTGGGTGTTAATTTGGAAGA	320
Consensus	taatgactctagctacctgggtgggtgttaatttggaaga	
HBC-3FLAG.CMV-F(H03)	TCCAGCATCTAGAGACCTAGTAGTCAGTTATGTCAACACT	399
HBC_ORF	TCCAGCATCTAGAGACCTAGTAGTCAGTTATGTCAACACT	360
Consensus	tccagcatctagagacctagtagtcagttatgtcaacact	
HBC-3FLAG.CMV-F(H03)	AATATGGGCCTAAAGTTCAGGCAACTCTTGTGGTTTCACA	439
HBC_ORF	AATATGGGCCTAAAGTTCAGGCAACTCTTGTGGTTTCACA	400
Consensus	aatatgggcctaaagttcaggcaactcttgtggtttcaca	
HBC-3FLAG.CMV-F(H03)	TTTCTTGTCTCACTTTTGGAAGAGAAACCGTTATAGAGTA	479
HBC_ORF	TTTCTTGTCTCACTTTTGGAAGAGAAACCGTTATAGAGTA	440
Consensus	tttcttgtctcacttttggaagagaaaccgttatagagta	
HBC-3FLAG.CMV-F(H03)	TTTGGTGTCTTTCGGAGTGTGGATTCGCACTCCTCCAGCT	519
HBC_ORF	TTTGGTGTCTTTCGGAGTGTGGATTCGCACTCCTCCAGCT	480
Consensus	tttggtgtctttcggagtgtggattcgcactcctccagct	
HBC-3FLAG.CMV-F(H03)	TATAGACCACCAAATGCCCCTATCCTATCAACACTTCCGG	559
HBC_ORF	TATAGACCACCAAATGCCCCTATCCTATCAACACTTCCGG	520
Consensus	tatagaccaccaaatgcccctatcctatcaacacttccgg	
HBC-3FLAG.CMV-F(H03)	AAACTACTGTTGTTAGACGACGAGGCAGGTCCCCTAGAAG	599
HBC_ORF	AAACTACTGTTGTTAGACGACGAGGCAGGTCCCCTAGAAG	560
Consensus	aaactactgttgttagacgacgaggcaggtcccctagaag	
HBC-3FLAG.CMV-F(H03)	AAGAACTCCCTCGCCTCGCAGACGAAGGTCTCAATCGCCG	639
HBC_ORF	AAGAACTCCCTCGCCTCGCAGACGAAGGTCTCAATCGCCG	600
Consensus	aagaactccctcgcctcgcagacgaaggtctcaatcgccg	
HBC-3FLAG.CMV-F(H03)	CGTCGCAGAAGATCTCAATCTCGGGAACCTCAATGT	675
HBC_ORF	CGTCGCAGAAGATCTCAATCTCGGGAACCTCAATGT	636
Consensus	cgtcgcagaagatctcaatctcgggaacctcaatgt	

**Table S1. The list of differentially expressed protein between HBc and control**		
**Gene names**	**Description**	
CDA	Cytidine deaminase	Downregulated
IFNGR1	Interferon gamma receptor 1	Downregulated
MVP	Major vault protein	Downregulated
RNF149	E3 ubiquitin-protein ligase RNF149	Downregulated
EPHA2	Ephrin type-A receptor 2	Downregulated
TAX1BP1	Isoform 3 of Tax1-binding protein 1	Downregulated
S100A11	Protein S100-A11	Downregulated
COTL1	Coactosin-like protein	Downregulated
TGFBR2	TGF-beta receptor type-2	Downregulated
FBLIM1	Filamin-binding LIM protein 1	Downregulated
KRT19	Keratin, type I cytoskeletal 19	Downregulated
APLP2	Isoform 5 of Amyloid-like protein 2	Downregulated
PRKAR1A	cAMP-dependent protein kinase type I-alpha regulatory subunit	Downregulated
HP	Haptoglobin (Fragment)	Downregulated
STX3	Isoform B of Syntaxin-3	Downregulated
SQSTM1	Isoform 2 of Sequestosome-1	Downregulated
NCOA4	Nuclear receptor coactivator 4 (Fragment)	Downregulated
GPR126	Isoform 2 of G-protein coupled receptor 126	Downregulated
AIM1	Absent in melanoma 1 protein	Downregulated
SLC2A1	Solute carrier family 2, facilitated glucose transporter member 1	Downregulated
UBE2C	Isoform 3 of Ubiquitin-conjugating enzyme E2 C	Downregulated
SERPINA1	Alpha-1-antitrypsin	Downregulated
PSAP	Proactivator polypeptide	Downregulated
CALCOCO2	Isoform 5 of Calcium-binding and coiled-coil domain-containing protein 2	Downregulated
NINJ1	Ninjurin-1	Downregulated
TACC1	Isoform 7 of Transforming acidic coiled-coil-containing protein 1	Downregulated
SDCBP	Syntenin-1	Downregulated
TMEM30A	Isoform 2 of Cell cycle control protein 50A	Downregulated
JAK1	Tyrosine-protein kinase JAK1	Downregulated
KRT23	Keratin, type I cytoskeletal 23	Downregulated
SNX17	Isoform 2 of Sorting nexin-17	Downregulated
SH3BGRL	SH3 domain-binding glutamic acid-rich-like protein	Downregulated
TRUB2	Probable tRNA pseudouridine synthase 2	Downregulated
RHOB	Rho-related GTP-binding protein RhoB	Downregulated
PPIC	Peptidyl-prolyl cis-trans isomerase C	Downregulated
SLC1A4	Isoform 2 of Neutral amino acid transporter A	Downregulated

RCN1	Reticulocalbin-1	Downregulated
TMEM2	Isoform 2 of Transmembrane protein 2	Downregulated
SLC7A2	Low affinity cationic amino acid transporter 2	Downregulated
ATP2B4	Isoform ZB of Plasma membrane calcium-transporting ATPase 4	Downregulated
PI4K2A	Phosphatidylinositol 4-kinase type 2-alpha	Downregulated
APOA2	Apolipoprotein A-II	Downregulated
SLC39A14	Zinc transporter ZIP14 (Fragment)	Downregulated
STXBP1	Syntaxin-binding protein 1	Downregulated
FGFR4	Fibroblast growth factor receptor	Downregulated
VAMP8	Vesicle-associated membrane protein 8	Downregulated
SPRY4	Protein sprouty homolog 4	Downregulated
S100A6	Protein S100-A6 (Fragment)	Downregulated
C1GALT1	Glycoprotein-N-acetylgalactosamine 3-beta-galactosyltransferase 1	Downregulated
GIPC1	PDZ domain-containing protein GIPC1	Downregulated
SH3BGRL3	SH3 domain-binding glutamic acid-rich-like protein 3	Downregulated
NPC1	Niemann-Pick C1 protein	Downregulated
SNX3	Isoform 4 of Sorting nexin-3	Downregulated
SLC30A1	Zinc transporter 1	Downregulated
SLC1A5	Neutral amino acid transporter B(0)	Downregulated
F11R	Junctional adhesion molecule A	Downregulated
KYNU	Kynureninase	Downregulated
ERBB3	Receptor tyrosine-protein kinase erbB-3	Downregulated
IGF2R	Cation-independent mannose-6-phosphate receptor	Downregulated
TCEB3	Transcription elongation factor B polypeptide 3	Downregulated
SFN	14-3-3 protein sigma	Downregulated
SLC38A2	Sodium-coupled neutral amino acid transporter 2	Downregulated
ECE1	Endothelin-converting enzyme 1	Downregulated
HLA-A	HLA class I histocompatibility antigen, A-69 alpha chain	Downregulated
SQRDL	Sulfide:quinone oxidoreductase, mitochondrial	Downregulated
FAT1	Protocadherin Fat 1	Downregulated
SERPINA3	Alpha-1-antichymotrypsin	Downregulated
TPT1	Translationally-controlled tumor protein	Downregulated
PBXIP1	Pre-B-cell leukemia transcription factor-interacting protein 1	Downregulated
LGALS3BP	Galectin-3-binding protein	Downregulated
GBA	Glucosylceramidase	Downregulated
LLGL1	Lethal(2) giant larvae protein homolog 1	Downregulated
ABCC2	Canalicular multispecific organic anion transporter 1	Downregulated
TACC2	Isoform 2 of Transforming acidic coiled-coil-containing protein 2	Downregulated
B2M	Beta-2-microglobulin	Downregulated
ITCH	Isoform 2 of E3 ubiquitin-protein ligase Itchy homolog	Downregulated

VASP	Vasodilator-stimulated phosphoprotein	Downregulated
GUF1	Translation factor GUF1, mitochondrial	Downregulated
CAPG	Macrophage-capping protein	Downregulated
KCTD10	BTB/POZ domain-containing adapter for CUL3-mediated RhoA degradation protein 3	Downregulated
OAT	Ornithine aminotransferase, mitochondrial	Downregulated
QTRT1	Queuine tRNA-ribosyltransferase	Upregulated
SORBS2	Isoform 9 of Sorbin and SH3 domain-containing protein 2	Upregulated
C11orf54	Ester hydrolase C11orf54 (Fragment)	Upregulated
CTSD	Cathepsin D	Upregulated
HNMT	Histamine N-methyltransferase	Upregulated
PHKB	Isoform 4 of Phosphorylase b kinase regulatory subunit beta	Upregulated
LRWD1	Leucine-rich repeat and WD repeat-containing protein 1	Upregulated
EPHX2	Isoform 2 of Bifunctional epoxide hydrolase 2	Upregulated
KTN1	Kinectin	Upregulated
FNBP1	Isoform 4 of Formin-binding protein 1	Upregulated
EPB49	Dematin	Upregulated
FTCD	Formimidoyltransferase-cyclodeaminase	Upregulated
AUH	Isoform 2 of Methylglutaconyl-CoA hydratase, mitochondrial	Upregulated
MYO1D	Unconventional myosin-Id	Upregulated
SAR1B	GTP-binding protein SAR1b	Upregulated
NFATC2	Isoform 3 of Nuclear factor of activated T-cells, cytoplasmic 2	Upregulated
POR	NADPH--cytochrome P450 reductase	Upregulated
PPP6R2	Isoform 6 of Serine/threonine-protein phosphatase 6 regulatory subunit 2	Upregulated
PGK1	Phosphoglycerate kinase 1	Upregulated
SUN2	SUN domain-containing protein 2	Upregulated
C7orf10	Isoform 4 of CaiB/baiF CoA-transferase family protein C7orf10	Upregulated
DDB2	DNA damage-binding protein 2	Upregulated
CBR4	Carbonyl reductase family member 4	Upregulated
GALK1	Galactokinase	Upregulated
SARDH	Sarcosine dehydrogenase	Upregulated
PECR	Peroxisomal trans-2-enoyl-CoA reductase	Upregulated
CPT1A	Carnitine O-palmitoyltransferase 1, liver isoform	Upregulated
PDCD4	Isoform 2 of Programmed cell death protein 4	Upregulated
RPS4Y1	40S ribosomal protein S4, Y isoform 1	Upregulated
FLNB	Isoform 8 of Filamin-B	Upregulated
CDK6	Cyclin-dependent kinase 6	Upregulated
ADH6	Alcohol dehydrogenase 6	Upregulated
PIPOX	Peroxisomal sarcosine oxidase	Upregulated
P4HA1	Isoform 3 of Prolyl 4-hydroxylase subunit alpha-1	Upregulated
CP	Ceruloplasmin (Fragment)	Upregulated

TF	Serotransferrin	Upregulated
DDAH2	N(G),N(G)-dimethylarginine dimethylaminohydrolase 2	Upregulated
RBM33	RNA-binding protein 33	Upregulated
KDM3A	Lysine-specific demethylase 3A	Upregulated
GBE1	1,4-alpha-glucan-branching enzyme	Upregulated
GLB1	Isoform 3 of Beta-galactosidase	Upregulated
LDHA	L-lactate dehydrogenase A chain	Upregulated
WBP4	WW domain-binding protein 4	Upregulated
KDM5C	Isoform 4 of Lysine-specific demethylase 5C	Upregulated
FBXO7	Isoform 3 of F-box only protein 7	Upregulated
PHYH	Phytanoyl-CoA dioxygenase, peroxisomal	Upregulated
PLIN2	Perilipin-2	Upregulated
FAM162A	Protein FAM162A	Upregulated
SEC24A	Protein transport protein Sec24A	Upregulated
AKR1C3	Aldo-keto reductase family 1 member C3	Upregulated
DAK	Bifunctional ATP-dependent dihydroxyacetone kinase/FAD-AMP lyase (cyclizing)	Upregulated
CISD3	CDGSH iron-sulfur domain-containing protein 3, mitochondrial	Upregulated
PEG3	Isoform 2 of Paternally-expressed gene 3 protein	Upregulated
DDC	Isoform 3 of Aromatic-L-amino-acid decarboxylase	Upregulated
SERPINB9	Serpin B9	Upregulated
AOX1	Aldehyde oxidase	Upregulated
PPFIBP2	Liprin-beta-2	Upregulated
ALDOC	Fructose-bisphosphate aldolase C	Upregulated
BCAP31	B-cell receptor-associated protein 31	Upregulated
APOL2	Apolipoprotein L2	Upregulated
SORBS1	Isoform 4 of Sorbin and SH3 domain-containing protein 1	Upregulated
ANXA13	Annexin A13	Upregulated
AKR1D1	Isoform 2 of 3-oxo-5-beta-steroid 4-dehydrogenase	Upregulated
IL1RN	Isoform 4 of Interleukin-1 receptor antagonist protein	Upregulated
BHMT2	Isoform 2 of S-methylmethionine--homocysteine S-methyltransferase BHMT2	Upregulated
SOD1	Superoxide dismutase [Cu-Zn]	Upregulated
GSTA4	Glutathione S-transferase A4	Upregulated
CKAP4	Cytoskeleton-associated protein 4	Upregulated
PDZK1	Na(+)/H(+) exchange regulatory cofactor NHE-RF3	Upregulated
CYP4F11	Cytochrome P450 4F11	Upregulated
AKR1B10	Aldo-keto reductase family 1 member B10	Upregulated
PTGR1	Isoform 2 of Prostaglandin reductase 1	Upregulated
NADKD1	NAD kinase 2, mitochondrial	Upregulated
CES1	Liver carboxylesterase 1	Upregulated
GNPAT	Dihydroxyacetone phosphate acyltransferase	Upregulated

EBAG9	Receptor-binding cancer antigen-expressed on SiSo cells	Upregulated
AGXT	Serine--pyruvate aminotransferase	Upregulated
AGPS	Alkyldihydroxyacetonephosphate synthase, peroxisomal	Upregulated
ALDH1L1	Cytosolic 10-formyltetrahydrofolate dehydrogenase	Upregulated
BHMT	Betaine--homocysteine S-methyltransferase 1	Upregulated
NDRG1	Protein NDRG1	Upregulated
ENTPD5	Ectonucleoside triphosphate diphosphohydrolase 5	Upregulated
PCK1	Phosphoenolpyruvate carboxykinase, cytosolic [GTP]	Upregulated
ALCAM	CD166 antigen (Fragment)	Upregulated
ARG1	Arginase-1	Upregulated

Table S2. NMR data for the metabolites assignment

Number	CAS No	Metabolites	δ ^{1}H and multiplicity	Proton groups	P-Value
1		Unknown1	0.90(s)	CH_3	0.84
			0.94(s)	CH_3	
2	73-32-5	Isoleucine	0.94 (t)	δ-CH_3	0.96
			1.01 (d)	γ'-CH_3	
			1.28(m)	half γ-CH_2	
			1.46 (m)	half γ-CH_2	
			1.98 (m)	β-CH	
			3.67 (dd)	α-CH	
3	61-90-5	Leucine	0.95 (d)	δ-CH_3	0.94
			0.96 (d)	δ′-CH_3	
			1.70(m)	γ-CH	
			1.72 (m)	β-CH_2	
			3.76 (d)	α-CH	
4	72-18-4	Valine	0.99 (d)	γ'-CH_3	0.96
			1.05 (d)	γ-CH_3	
			2.27 (m)	β-CH	
			3.61 (d)	α-CH	
5	79-33-4	Lactate	1.33 (d)	β-CH_3	0.92
			4.12 (q)	α-CH	
6	72-19-5	Threonine	1.33 (d)	γ-CH_3	0.98
			3.58 (d)	α-CH	
			4.26 (m)	β-CH_2	
7	56-87-1	Lysine	1.44 (m)	Half -CH_2	0.87
			1.49 (m)	Half -CH_2	
			1.72 (m)	γ-CH_2	
			1.92 (m)	β-CH_2	
			3.04 (t)	-CH_2	
			3.74 (t)	α-CH	
8	56-41-7	Alanine	1.48(d)	β-CH_3	0.98
			3.80 (q)	α-CH	
9		N-acetylated glycoproteins	2.02 (s)	CH_3	0.79
10	7512-17-6	GlcNAc	2.08 (s)	NA-H	-0.66
			3.46	α-C4H	
			3.46	β-C4,5H	
			3.53	β-C3H	
			3.67	β-C2H	
			3.76	α-C3H	
			4.71	β-C1H	
			3.85 (m)	α-C2,5,6H	
			5.21(d)	α-C1H	

11	6893-26-1	Glutamate	2.08(m)	β-CH_2	0.84
			2.35(m)	γ-CH_2	
			3.76(m)	α-CH	
12	70-18-8	Glutathione	2.16(m)	Glu β	0.96
			2.56(m)	Glu γ	
			2.95(m)	Cys β	
			3.78(t)	Glu α	
			4.59(dd)	Cys α	
13	110-15-6	Succinate	2.41(s)	CH_2	0.94
14	56-84-8	Aspartate	2.69(dd)	β-CH	0.82
			2.81(dd)	β'-CH	
			3.90(m)	α-CH	
15		Unknown2	2.98(m)		0.96
			3.34(t)		
			4.79		
16	57-00-1	Creatine	3.04(s)	CH_3	0.95
			3.93(s)	CH_2	
17	60-18-4	Tyrosine	3.08	β-CH_2	0.86
			3.95(dd)	α-CH	
			6.91(d)	C3,5H,ring	
			7.20(d)	C2,6H,ring	
18	62-49-7	Choline	3.21(s)	N-CH_3	0.73
			3.56(m)	β-CH_2	
			3.99(m)	α-CH_2	
19	3616-04-4	Phosphocholine	3.22(s)	N-CH3	0.97
			3.60(m)	α-CH_2	
			4.17(m)	β-CH_2	
20	492-61-5	β-Glucose	3.24 (m)	2-CHOH	0.94
			3.39 (t)	4-CHOH	
			3.47 (m)	5-CHOH	
			3.48 (t)	3-CHOH	
			3.72 (m)	half-CH_2OH	
			3.89 (dd)	half-CH_2OH	
			4.64 (d)	1-CHOH	
21	87-89-8	*myo*-Inositol	3.26(t)	C5H	0.36
			3.54(dd)	C1,3H	
			3.62(t)	C4,6H	
			4.06(t)	C2H	
22	107-35-7	Taurine	3.27(t)	N-CH_2	0.55
			3.43(t)	S-CH_2	
23	492–62–6	α-Glucose	3.40 (t)	4-CHOH	0.67
			3.53 (dd)	2-CHOH	
			3.71 (t)	3-CHOH	
			3.75 (m)	half-CH_2OH	

			3.83 (m)	half-CH_2OH	
			3.83 (m)	5-CHOH	
			5.23 (d)	1-CHOH	
24	812-00-0	Monomethylphosphate	3.47(d)	CH_3	0.34
25	28053-08-9	UDP-Glc	3.47	G5-H	0.81
			3.54	G4-H	
			3.77	G3-H	
			3.90	G2-H	
			3.86/3.78	G6-H	
			4.26/4.21(m)	C5'H,ribose	
			4.29(m)	C4',ribose	
			4.38(m)	C2'3'H,ribose	
			5.61	G1-H	
			5.98(d)	C5,ring	
			5.99(d)	C1'H,ribose	
			7.96(d)	C6,ring	
26	63700-19-6	UDP-GlcA	3.51(dd)	G3-H	0.97
			3.59(m)	G4-H	
			3.79(dd)	G2-H	
			4.14(dd)	G5-H	
			4.25/4.19	C5'H,ribose	
			4.29(m)	C4'H,ribose	
			4.38(m)	C2'3'H,ribose	
			5.62	G1-H	
			5.98(d)	C5,ring	
			6.00(d)	C1'H,ribose	
			7.95(d)	C6,ring	
27	56-40-6	Glycine	3.56(s)	α-CH_2	0.92
28	28319-77-9	Glycerolphosphocholine	3.60(dd)	1-CH_2	0.98
			3.89(m)	2-CH	
			3.77(dd)	3-CH_2	
			4.32(t)	α-CH_2	
			3.68(t)	β-CH_2	
			3.23(s)	N-CH_3	
29	118-00-3	Guanosine	3.84	CH5-ribose	0.72
			4.24(dt)	CH4-ribose	
			4.41(dd)	CH3-ribose	
			5.92(d)	CH1-ribose	
			8.00(s)	CH-ring	
30	58-63-9	Inosine	3.87(dd)	CH5-ribose	0.89
			4.28(dt)	CH4-ribose	
			4.44(dd)	CH3-ribose	
			4.78(dd)	CH2-ribose	
			6.10(d)	CH1-ribose	

			8.24(s)	CH-ring	
			8.35(s)	CH-ring	
31	91183-98-1	UDP-GlcNAc	3.87	G6-H	0.74
			3.93	G5-H	
			2.08(s)	NA-H	
			3.55(dd)	G4-H	
			3.82(m)	G3-H	
			3.99(m)	G2-H	
			4.25/4.19(m)	C5’H,ribose	
			4.29(m)	C4’H,ribose	
			4.37(m)	C2’3’H,ribose	
			5.52(dd)	G1-H	
			5.97(d)	C5,ring	
			5.98(d)	C1’H,ribose	
			7.96(d)	C6,ring	
32	108320-87-2	UDP-GalNAc	3.78	G6-H	-0.73
			3.79	G5-H	
			2.09(s)	NA-H	
			3.76(m)	G4-H	
			3.97(dd)	G3-H	
			4.05(m)	G2-H	
			4.25/4.19(m)	C5’H,ribose	
			4.29(m)	C4’,ribose	
			4.37(m)	C2’3’H,ribose	
			5.55(dd)	G1-H	
			5.97(d)	C5,ring	
			5.99(d)	C1’H,ribose	
			7.96(d)	C6,ring	
33	58-64-0	ADP	4.01(m)	C5H-ribose	0.66
			4.38(m)	C4H-ribose	
			4.51(m)	C3H-ribose	
			4.61(m)	C2H-ribose	
			6.14(d)	C1H-ribose	
			8.27(s)	CH-ring	
			8.54(s)	CH-ring	
34	74431-23-5	IMP	4.03(m)	CH5-ribose	0.32
			4.77	CH2-ribose	
			4.37(dt)	CH4-ribose	
			4.51(dd)	CH3-ribose	
			6.14(d)	CH1-ribose	
			8.22(s)	CH-ring	
			8.61(s)	CH-ring	
35	58-96-8	Uridine	4.12(q)	C5’H,ribose	0.44
			4.23(t)	C4’H,ribose	

			4.34(t)	C3’H,ribose	
			5.90(d)	C5,ring	
			5.92(d)	C1’H,ribose	
			7.88(d)	C6,ring	
36	53-84-9	NAD	4.21/4.24	A5’H2	0.67
			4.23/4.36	N5’H2	
			4.37	A2’H	
			4.43	N3’H	
			4.48	N2’H	
			4.51	A3’H	
			4.55	N4’H	
			6.04(d)	A1’H	
			6.09(d)	N1’H	
			8.18(s)	A2Hring	
			8.20(m)	N5ring	
			8.43(s)	A8H ring	
			8.83(d)	N4ring	
			9.15(d)	N6ring	
			9.34(s)	N2ring	
37	58-98-0	UDP	4.23(dd)	CH5-ribose	0.28
			4.27(m)	CH4-ribose	
			4.39(t)	CH2-ribose	
			4.43(t)	CH3-ribose	
			5.96(broad, s)	CH1-ribose	
			5.98(d)	CH-ring	
			8.00(d)	CH-ring	
38	66-22-8	Uracil	5.80(d)	C5H	0.30
			7.54(d)	C6H	
39	110-17-8	Fumarate	6.52(s)	C2,3H	0.82
40	71-00-1	Histidine	7.10(s)	C4H,ring	0.33
			7.86(s)	C2H,ring	
41	63-91-2	Phenylalanine	7.33(m)	C2,6,ring	0.94
			7.38(m)	C4,ring	
			7.42(m)	C3,5,ring	
42	73-22-3	Tryptophan	7.55(d)	C7H,ring	0.73
			7.74(d)	C4H,ring	
43	64-18-6	Formate	8.46 (s)	HCOO	0.76

Note: s, singlet; d, doublet; t, triplet; q, quartet; m, multiplet; b, broad peak; dd, doublet of doublets

Table S3.The list the interacting protein of HBc

Gene names	Intensity 3Flag-1	Intensity HBC-1	LOG10-3Flag-1	LOG10-HBC-1	Intensity 3Flag-2	Intensity HBC-2	LOG10-3Flag-2	LOG10-HBC-2	HBC1-FLAG1	HBC2-FLAG2
HBCORF	0	145530000	0.00	8.16	0	122060000	0.00	8.09	8.16	8.09
ALDOB	0	32049000	0.00	7.51	0	13401000	0.00	7.13	7.51	7.13
ASS1	0	62940000	0.00	7.80	0	6821500	0.00	6.83	7.80	6.83
GDI2	0	517140	0.00	5.71	0	4711900	0.00	6.67	5.71	6.67
MDH1	0	2382700	0.00	6.38	0	4514800	0.00	6.65	6.38	6.65
BHMT	0	11231000	0.00	7.05	0	4467700	0.00	6.65	7.05	6.65
PSAT1	0	1038800	0.00	6.02	0	3908100	0.00	6.59	6.02	6.59
HIST1H1C	0	4264800	0.00	6.63	0	3884400	0.00	6.59	6.63	6.59
ALDH1L1	0	7595700	0.00	6.88	0	3509200	0.00	6.55	6.88	6.55
SARDH	0	8760300	0.00	6.94	0	2866100	0.00	6.46	6.94	6.46
ASL	0	7761800	0.00	6.89	0	2385900	0.00	6.38	6.89	6.38
NQO1	0	2197700	0.00	6.34	0	2344000	0.00	6.37	6.34	6.37
OTC	0	6147100	0.00	6.79	0	1707900	0.00	6.23	6.79	6.23
KRT13	0	1183000	0.00	6.07	0	1523500	0.00	6.18	6.07	6.18
NME1	0	845770	0.00	5.93	0	1479800	0.00	6.17	5.93	6.17
GNMT	0	5466600	0.00	6.74	0	1456000	0.00	6.16	6.74	6.16
MLX	0	1102900	0.00	6.04	0	1403700	0.00	6.15	6.04	6.15
CYB5A	0	6275900	0.00	6.80	0	1055300	0.00	6.02	6.80	6.02
SSB	0	270230	0.00	5.43	0	1051400	0.00	6.02	5.43	6.02
CA3	0	3528800	0.00	6.55	0	1039300	0.00	6.02	6.55	6.02
ALDH6A1	0	2625100	0.00	6.42	0	1039300	0.00	6.02	6.42	6.02
UROC1	0	1179900	0.00	6.07	0	1038300	0.00	6.02	6.07	6.02
HPD	0	3251800	0.00	6.51	0	1027400	0.00	6.01	6.51	6.01
EIF3M	0	30215	0.00	4.48	0	885050	0.00	5.95	4.48	5.95
HMGCS2	0	3620900	0.00	6.56	0	860500	0.00	5.93	6.56	5.93
GOT1	0	3046500	0.00	6.48	0	851710	0.00	5.93	6.48	5.93
PAICS	0	341650	0.00	5.53	0	814660	0.00	5.91	5.53	5.91
ANXA11	0	79907	0.00	4.90	0	730230	0.00	5.86	4.90	5.86
DAK	0	2075400	0.00	6.32	0	631470	0.00	5.80	6.32	5.80

FBXO2	0	81178	0.00	4.91	0	578720	0.00	5.76	4.91	5.76
ARF1	0	1322900	0.00	6.12	0	559030	0.00	5.75	6.12	5.75
PYGL	0	3411700	0.00	6.53	0	556110	0.00	5.75	6.53	5.75
ACTR2	0	335310	0.00	5.53	0	552960	0.00	5.74	5.53	5.74
HSPE1	0	152930	0.00	5.18	0	527130	0.00	5.72	5.18	5.72
GART	0	499170	0.00	5.70	0	515660	0.00	5.71	5.70	5.71
SEC31A	0	28299	0.00	4.45	0	493220	0.00	5.69	4.45	5.69
ALDH4A1	0	1692300	0.00	6.23	0	482710	0.00	5.68	6.23	5.68
RPS4Y1	0	1513200	0.00	6.18	0	456880	0.00	5.66	6.18	5.66
RPS6KA3	0	88319	0.00	4.95	0	453220	0.00	5.66	4.95	5.66
MGST1	0	2304000	0.00	6.36	0	425760	0.00	5.63	6.36	5.63
RGN	0	489560	0.00	5.69	0	392550	0.00	5.59	5.69	5.59
GNPNAT1	0	689190	0.00	5.84	0	364110	0.00	5.56	5.84	5.56
EIF2B4	0	52792	0.00	4.72	0	343230	0.00	5.54	4.72	5.54
DECR1	0	717140	0.00	5.86	0	333020	0.00	5.52	5.86	5.52
SERPINA7	0	123100	0.00	5.09	0	316300	0.00	5.50	5.09	5.50
ACSL1	0	2523300	0.00	6.40	0	275340	0.00	5.44	6.40	5.44
ACTN1	0	680670	0.00	5.83	0	253280	0.00	5.40	5.83	5.40
NDUFA4	0	870930	0.00	5.94	0	240370	0.00	5.38	5.94	5.38
APRT	0	229360	0.00	5.36	0	237930	0.00	5.38	5.36	5.38
MCM5	0	306880	0.00	5.49	0	229330	0.00	5.36	5.49	5.36
ATP5O	0	2216000	0.00	6.35	0	226120	0.00	5.35	6.35	5.35
ANPEP	0	1780300	0.00	6.25	0	221350	0.00	5.35	6.25	5.35
DCXR	0	380060	0.00	5.58	0	191430	0.00	5.28	5.58	5.28
CASP3	0	205370	0.00	5.31	0	182150	0.00	5.26	5.31	5.26
SNRPD2	0	115180	0.00	5.06	0	180010	0.00	5.26	5.06	5.26
CTSB	0	597910	0.00	5.78	0	158950	0.00	5.20	5.78	5.20
MTCH2	0	183350	0.00	5.26	0	157310	0.00	5.20	5.26	5.20
NDRG2	0	190920	0.00	5.28	0	156130	0.00	5.19	5.28	5.19
DAD1	0	50090	0.00	4.70	0	153770	0.00	5.19	4.70	5.19
DIS3	0	35672	0.00	4.55	0	117960	0.00	5.07	4.55	5.07
SRP19	0	146910	0.00	5.17	0	114220	0.00	5.06	5.17	5.06

ACOX1	0	538340	0.00	5.73	0	113350	0.00	5.05	5.73	5.05
DSTN	0	75271	0.00	4.88	0	103150	0.00	5.01	4.88	5.01
SHMT1	0	194580	0.00	5.29	0	99368	0.00	5.00	5.29	5.00
H1F0	0	349740	0.00	5.54	0	85276	0.00	4.93	5.54	4.93
PPP1CC	0	385970	0.00	5.59	0	69033	0.00	4.84	5.59	4.84
GLG1	0	938180	0.00	5.97	0	64927	0.00	4.81	5.97	4.81
PAH	0	888920	0.00	5.95	0	42250	0.00	4.63	5.95	4.63

Table S4. Primer Sequences for ChIP

Gene	Primer number	ChIP primer F	ChIP primer R	MLX binding motif
**CES1**	CES1-1	GCTGTCAGAGCATCCTCATTCC	TGCCCAAGGCCATGAGTTGTAG	CACGTG
	CES1-2	TGGGTACTTGGATGGATGGATG	CATCGGTGTCAGAGAACTCACT	TGATAA
**NPC1**	NPC1-1	ACTCGCGGATCGTCACA	CTTCTCCTCCCAACCCAGTC	CGGGCT
	NPC1-2	CTCAGCGTAATTGTCGGAGCC	GCGAGGTTTCAGCGAAGGGT	CCGGAAGT
**AKR1D1**	AKR1D1-1	ATGCTGAGTGAAAGAAGCCAATCTC	GAACTCTATCCTATCACACCCTCCC	TGATAA
	AKR1D1-2	GCCATGTTATTGTTCCTCTAGTCCA	AGCAGGACTGGAAAGGGATCTAGG	TGATAA
**PCK1**	PCK1-1	ACTGGAACTGTCTATGTGGCACTT	CCCACCCTGATGTTAGAGTATTTGG	TGATAA
	PCK1-2	TGTTTCTTAGGCAAGATAGCTCCAT	TGCCACAACAGATAGAGAGACCAT	CACGTG
	PCK1-3	ATCCTGAAAGACACAACTGGCTGAA	GGCGTGGGTCACACATCATCAA	TGATAA
**PSAP**	PSAP-1	GGATTACAGGAGTGAGCCAGCAT	AAGTAGTCGTGTAACAGGGTACAGG	CACGTG
**SORBS1**	SORBS1-1	GCCAGGACGGAAAGGGTTACTAGG	CGGATGGGAGGCGAAGAAGTGT	CGGGCTC
**AGPS**	AGPS-1	TGTTCTATTTCCTGACCTAGATGGT	TCTGAGCTTCTGAGTGATCCCTAAA	TGATAA
	AGPS-2	GCCTACTGGGAGTAACAGACATGA	TCCCTGAGAATTTATCCTCTTGCTT	TGATAA
	AGPS-3	TTCTTATGCAAGACACCGGCTGAA	CCAAGAGCTGTGAAGACATCTCAGT	CACGTG
**GNPAT**	GNPAT-1	TCACTGGAGGTCTAGAACTTGAATA	AAAGGACTCACAGGACCCAAGA	TGATAA
	GNPAT-2	AGGACGGGCAACACAGGAGA	TCCGACACTTCTTGACGCTCTG	CACGTG
**ALDOC**	ALDOC-1	TCAGTGCTGATGACCGTGTGAA	CGATGCCCTTATCCTGGATGGTT	TGATAA

**Table S5. Primer Sequences for Real-time PCR**		
**Gene**	**Sense Primer**	**Anti-sense Primer**
ACTB	TGGTGGGCATGGGTCAGAAGGA	GGAGCCACACGCAGCTCATTGT
AGPS	AGGGAAGGAATGTTTGAGCGA	GCAGGACACATCAGGCCAT
AKR1C3	ACCCAGTCCTTTGTGCCTTGGC	ACGTTCTGTCTGATGCGCTGCT
AKR1D1	CACTTGGGAGGCGATGGAAGCT	ACCTGGTTGCTGACTGGCTTGT
ALDOC	ATGCCTCACTCGTACCCAG	TTTCCACCCCAATTTGGCTCA
CES1	GCGCTGGGTCCAGGACAACATT	ATGGCCCGGTGGAAGAGGTTCT
GNPAT	GCTGCTACGAATGTCGGGT	TGTCCCTTCGAGGAAAAATTCAA
NPC1	GCACCTTTTACCATCACTCCTG	GGCCACAGACAATAGAGCAGT
PCK1	TTGAGAAAGCGTTCAATGCCA	CACGTAGGGTGAATCCGTCAG
PSAP	ATGCAAAGACGTTGTCACCG	GGGAGGTAGGAGTCCACTATCT
SORBS1	CACAATCGAGAACAGCAAAAACG	ACCCGCCTACTGTCATCCTTT
